# Breaking barriers: how generic drugs democratize mental health

**DOI:** 10.3389/fphar.2025.1733732

**Published:** 2025-11-27

**Authors:** Gilberto Uriel Rosas-Sánchez, Marisol Mercado-Serna, Rafael Fernández-Demeneghi, María Isabel Pérez-Vega, Cesar Soria-Fregozo

**Affiliations:** 1 Departamento de Ciencias de la Tierra y de la Vida, Centro Universitario de Los Lagos, Universidad de Guadalajara, Lagos de Moreno, Jalisco, Mexico; 2 Programa de Estancias Posdoctorales por México, SECIHTI, Centro Universitario de Los Lagos, Universidad de Guadalajara, Lagos de Moreno, Jalisco, Mexico; 3 Instituto de Investigaciones en Comportamiento Alimentario y Nutrición, Universidad de Guadalajara, Ciudad Guzmán, Mexico

**Keywords:** anxiety, depression, generic drug, mental health, bioequivalence

## 1 Introduction

Mental health has become one of the greatest health crises of the 21st century. Current data show that anxiety and depression disorders have increased alarmingly, especially after the COVID-19 pandemic, affecting millions of people worldwide (Cipriani et al., 2018; Kupcova et al., 2023). Paradoxically, while the demand for psychiatric treatments is rising exponentially, access to effective medications remains limited by significant economic barriers (Xu et al., 2025; Ali et al., 2023). This situation raises a fundamental ethical dilemma: how can we ensure that pharmacological treatment for anxiety and depression is accessible to all socioeconomic groups? Generic medications are emerging as a tangible and scientifically supported solution to close this inequality gap. However, myths and resistance persist, limiting their widespread adoption (Rana and Roy, 2015; Dunne and Dunne, 2015). This article argues that generic drugs are not only a viable economic alternative but also an essential tool for democratizing access to mental health care without compromising therapeutic quality.

## 2 Patent administration of drugs

The administration of drug patents constitutes the legal framework that regulates pharmaceutical intellectual property rights, balancing the promotion of innovation with access to affordable medicines (Kannappan et al., 2021). A pharmaceutical patent grants exclusive rights to novel, non-obvious, and useful inventions, including active ingredients, formulations, manufacturing processes, or methods of use (Warchol, 2019). This system enables innovative companies to recover investments in research, development, and clinical trials (Warchol, 2019).

Although protection lasts 20 years from the filing date, the effective exclusivity period varies due to development time and regulatory approval (Song and Han, 2016). The Hatch-Waxman Act in the U.S. establishes mechanisms to extend patent terms and facilitate generic drug approval (Kannappan et al., 2021; Sokal and Gerstenblith, 2010). The end of exclusivity, known as the “patent cliff,” results in losses of millions of dollars for innovators due to generic competition (Kakkar, 2015). Generic drugs are bioequivalent to brand-name drugs, releasing the same amount of active ingredient in the same period (Chow, 2014; Holman, 2019; Soufsaf et al., 2022), allowing them to be marketed at significantly lower prices for people who lack the resources to purchase a patented drug (Holman, 2019; Nguyen et al., 2025).

## 3 Perception *versus* reality: the therapeutic equivalence of generics

One of the most significant obstacles to the widespread adoption of generic medications in mental health is the misperception that they are a “second-class” option (Dunne and Dunne, 2015; Colgan et al., 2015). This belief lacks a solid scientific foundation and contradicts decades of rigorous regulatory evidence. Generic medications must demonstrate bioequivalence to their brand-name counterparts, meaning they must have the same bioavailability and produce equivalent clinical effects (Borgheini, 2003; Pettersen et al., 2022). Bioequivalence criteria allow for variability in maximum plasma concentration (Cmax) and area under the curve (AUC) ranging from −20% to +25% between generic and reference products (Andrade, 2015; Peterson, 2011). This variability, which may seem concerning at first, falls within acceptable margins also observed between different batches of the same brand-name medication (Lechat et al., 2023). Scientific research has consistently shown that generic antidepressants, including fluoxetine, sertraline, and escitalopram, maintain efficacy and safety profiles comparable to their original versions (Solem et al., 2016). A study in Taiwan examining a large population-based cohort found that patients treated with generic versions of certain antidepressants (sertraline, paroxetine, escitalopram, venlafaxine, mirtazapine, and bupropion) had a higher risk of psychiatric hospitalization compared to those treated with their brand-name equivalents (Hsu et al., 2020). This suggests that for some drugs, brand-name versions might offer more protective effects on psychiatric hospitalization outcomes for depressive patients (Hsu et al., 2020). Another factor that can influence perceived efficacy is patient psychology. “Brand-name worship” and expectation psychology have been observed to impact antidepressant efficacy, where a patient’s belief in the superiority of a brand-name drug can lead to a perceived improvement in symptoms after switching from a generic to a brand-name counterpart, even if bioequivalence is established (Cai et al., 2013). Population-based cohort studies have shown that generic versions of cardiovascular and metabolic medications maintain clinical effectiveness comparable to their brand-name counterparts (Tian et al., 2020). International regulatory agencies, including the Food and Drug Administration (FDA) and European Medicines Agency (EMA), maintain identical quality standards for generic and brand-name medications. These standards range from the purity of the active ingredients to the manufacturing conditions, ensuring that patients receive a therapeutically equivalent product regardless of the brand (van der Meersch et al., 2011). Table 1 describes the bioequivalence and efficacy studies of brand-name and generic anxiolytics and antidepressants in clinical studies.

**TABLE 1:** Bioequivalence and efficacy studies of brand-name vs. generic anxiolytic and antidepressant drugs.

Drug	Study type	Key findings regarding bioequivalence and efficacy	References
Clonazepam	Clinical	A bioequivalence study of two oral tablet formulations (test/generic and reference/brand) of clonazepam 2 mg in healthy Mexican volunteers found similar rates and extent of absorption, and a consistent safety and tolerability profile, indicating bioequivalence	(Genis-Najera and Sañudo-Maury, 2024)
Diazepam	Clinical	Found bioinequivalence between a generic brand of diazepam and brand-name Valium, with the generic showing significantly slower absorption and lower peak plasma concentrations, potentially leading to therapeutic inequivalence	(Locniskar et al., 1989)
Escitalopram Oxalate	Clinical	A study comparing two formulations (generic and brand-name) of escitalopram oxalate 20 mg tablets in a healthy Chinese population confirmed bioequivalence under both fasting and fed conditions	(Li et al., 2020)
Bupropion XL 300 mg	Clinical	A prospective, randomized, double-blinded crossover clinical trial found no significant differences in peak plasma concentration and area under the plasma concentration-time curve (AUC0–24) for racemic bupropion or its major metabolites between the brand and three generic bupropion XL 300 mg products, indicating both bioequivalence and therapeutic equivalence	(Kharasch et al., 2019)
Multiple Antidepressants (e.g., Sertraline, Paroxetine, Escitalopram, Venlafaxine, Mirtazapine, Bupropion)	Clinical	A nationwide population-based study in Taiwan revealed that patients treated with generic versions of these antidepressants had a higher risk of psychiatric hospitalization compared to those treated with their brand-name counterparts, suggesting brand-name drugs may offer more protective effects against hospitalization for depression	(Hsu et al., 2020)
Sertraline	Clinical (Efficacy/Safety study)	A study investigating the risk of hospitalized depression and intentional self-harm with brand and authorized generic sertraline found no significant difference in hospitalization risk or intentional self-harm between brand and generic users after propensity score matching	(Pennap et al., 2022)
Venlafaxine	Clinical (Case report/Observational study)	A case report suggested that “brand-name worship and expectation psychology” might influence a patient’s perceived improvement when switching from generic to brand-name venlafaxine Another observational study explored patient adherence to and outcomes of generic *versus* brand-name venlafaxine for Generalized Anxiety Disorder	(Cai et al., 2013; Sicras-Mainar et al., 2015)
Various Anxiolytics and Antidepressants	Clinical and regulatory (Reviews/Systematic Reviews)	Regulatory approval for generic drugs typically requires demonstration of bioequivalence, which implies “essential similarity” to the original drug. However, several reviews highlight that bioequivalence does not always equate to therapeutic effectiveness, especially for psychotropic medications. Concerns about the true equivalence of generics to brand-name medications persist despite regulatory standards	(Borgheini, 2003; Desmarais et al., 2011; Cessak et al., 2016; Bałkowiec-Iskra et al., 2015; Rokita et al., 2015)

Considering the above, it is important to highlight that the bioequivalence clinical trials for generic drugs, Quality by Design (QbD) is a systematic, risk-based approach to pharmaceutical development that ensures consistent quality through a comprehensive understanding of the product and process from the outset, distinguishing it from conventional trial-and-error methods (Duarte et al., 2025). This approach aligns with ICH E6 (R3) guidance, which promotes Risk-Based Quality Management (RBQM) by focusing resources on activities critical to data integrity and patient safety (Dirks et al., 2024). Key elements include the Target Product Profile, Critical Quality Attributes, Design Space, and Control Strategy, which together optimize the safety, efficacy, and quality of the drug (Simões et al., 2024).

In bioequivalence studies, which are essential for demonstrating that generic drugs perform similarly to the reference drug (Fernandes et al., 2024), the application of QbD transforms quality assurance and control into proactive and preventive strategies. This involves proactive planning with thorough risk assessment, risk-based monitoring that focuses on critical data, and the use of Quality Tolerance Limits (QTLs) as a key control tool (Yan et al., 2025). The integration of analytical QbD ensures robust and continuously validated methods (Verch et al., 2022), while appropriate statistical applications ensure adequate sample sizes and pharmacokinetic analyses that meet established bioequivalence criteria, such as a 90% confidence interval between 80% and 125% (Hammami et al., 2017).

## 4 Real socioeconomic impact

The economic impact of generic medicines on the healthcare system is both indisputable and transformative (Ogbeta et al., 2024). Using generics instead of brand-name medicines provides bioequivalent alternatives to listed reference medicines (RLDs) with the same active ingredients, at 80%–85% lower cost than brand-name medicines (Straka et al., 2017). This substantial cost reduction represents the difference between access and exclusion for millions of patients. Brand-name medicines are typically 30%–60% more expensive than their generic counterparts, generating a significant impact on therapeutic accessibility (Rokita et al., 2015). Data from a global company that provides advanced analytics, technology solutions, and clinical research services to the life sciences and healthcare industries (IQVIA) show that generic and biosimilar medicines generated a record $133 billion in savings for patients and the healthcare system. These savings are not merely statistical; these benefits translate into lives saved and continued treatment that would otherwise be interrupted due to financial constraints (Gunnam et al., 2025).

In the context of mental health, where treatments often require long-term drug therapy, the cost difference is even more pronounced (Juraszek et al., 2024). A patient treated with generic sertraline can save between $200 and $400 annually compared to the brand-name version, enabling sustained therapeutic adherence, which is crucial for treatment success (Polsky et al., 2002). Therapeutic adherence, a critical factor in treating psychiatric disorders, improves significantly when costs are affordable (Barbui and Conti, 2015). Longitudinal studies have shown that patients who use generic medications have 30%–40% lower drug discontinuation rates than those limited to brand-name versions (Shrank et al., 2006; Choudhry et al., 2016). Comparative effectiveness research has shown that generic medications produce equivalent clinical outcomes, even when controlling for the nocebo effect associated with negative perceptions of generics (Desai et al., 2019).

## 5 Current challenges and resistances

Despite robust scientific evidence and demonstrated economic benefits, significant barriers to optimal adoption of generic medications in mental health remain (Kaplan et al., 2012; Alqawasmeh et al., 2025). These barriers exist at multiple levels of the healthcare system and reflect a complex interplay of psychological, commercial, and educational factors. Prescription resistance is one of the most prominent obstacles. Many healthcare professionals maintain unconscious biases toward brand-name medications, influenced by decades of aggressive pharmaceutical marketing and the misconception that “more expensive equals better quality” (Kaplan et al., 2012; Dunne et al., 2013). This preference is inadvertently passed on to patients, perpetuating distrust of generic alternatives. Bioequivalence concerns persist even when generic medications meet strict regulatory standards (Bate et al., 2016). Patients also experience understandable anxiety about changing medications they perceive as effective. In mental health, where symptom stability is particularly fragile, any therapeutic change raises legitimate concerns. However, these concerns are often based on incomplete information about the therapeutic equivalence of generics.

The pharmaceutical industry employs sophisticated strategies to preserve market share for brand-name drugs, including campaigns that subtly discredit generic alternatives and financial assistance programs that create temporary dependence on specific products. Most medications currently prescribed to treat schizophrenia, mood disorders, and anxiety are no more effective than the first generation of psychiatric medications introduced over 50 years ago (Giliberto et al., 2024; Paul and Potter, 2024). Projections indicate that pharmaceutical spending will continue to grow by 10%–12% annually, making the adoption of generic alternatives even more critical (Tichy et al., 2024). Poor health education about generic medications is another significant barrier. Both professionals and patients often lack up-to-date information on bioequivalence regulations, manufacturing processes, and scientific evidence supporting the efficacy of generics (Colgan et al., 2015; Shrank et al., 2011).

## 6 Discussion

The findings in this opinion piece reveal a persistent paradox in mental healthcare: although strong scientific evidence supports the bioequivalence and effectiveness of generic medications, their widespread adoption faces multiple barriers that perpetuate inequities in access to treatment. This discrepancy between evidence and clinical practice requires a detailed examination of its implications for contemporary healthcare systems. Resistance to generic substitution in psychiatry has unique characteristics compared to other medical specialties. A recent study on medication safety in patients with mental disorders in primary care found that a fragmented understanding of therapeutic challenges significantly contributes to the underuse of generic alternatives (Ayre et al., 2023). This fragmentation occurs at both the professional and patient levels, creating a vicious cycle of mistrust that affects therapeutic adherence regardless of the type of medication prescribed.

A critical aspect that emerges from the analysis is the differential impact of economic barriers on vulnerable populations. Research shows that low-income individuals often adopt concerning coping strategies, sacrificing basic needs to afford medications (Rohatgi et al., 2021). This issue is especially alarming in mental health, where therapeutic continuity is essential to prevent relapses and hospitalizations. Generic medications, by reducing costs by over 80%, could substantially mitigate this ethical dilemma, allowing patients to maintain effective treatments without compromising their food or housing security. The implementation of generic substitution policies presents additional complexities that require strategic attention. A multi-country study of pharmacists’ attitudes toward generic substitution policies found that professional acceptance varies significantly depending on the local regulatory and educational context (Babar et al., 2010). In systems where pharmacists received specific training on bioequivalence and effective communication with patients, generic substitution rates improved substantially. This suggests that educational interventions targeting health professionals are essential to maximize the potential of generics in mental health.

The patient perspective on generic substitution requires special consideration. Recent research on patient preferences for generic substitution policies shows that acceptance depends critically on the quality of information received and trust in the prescriber (Zhang et al., 2024). Patients who understood bioequivalence mechanisms and regulatory safeguards demonstrated significantly higher acceptance of generic alternatives. This finding highlights the importance of investing in health education campaigns to dispel misconceptions about the quality and efficacy of generics. Systemic barriers to optimal implementation of generic medications in mental health also include aspects related to institutional formularies and reimbursement policies. Studies on the effects of state regulations on generic substitution show that permissive legal frameworks, combined with appropriate financial incentives for prescribers and pharmacists, lead to significantly higher adoption rates (Shrank et al., 2010). However, regulatory differences across jurisdictions create geographic inequities in access to affordable therapeutic options.

An emerging challenge in discussions about generic drugs is the nocebo effect, documented in studies where patients who knew they were receiving generics reported lower perceived efficacy despite objective bioequivalence (Faasse and Petrie, 2013). This psychological phenomenon complicates the transition from brand-name to generic drugs, especially in conditions where symptoms are subjective and self-monitoring plays a central role. Physician-patient communication strategies that emphasize therapeutic equivalence without causing anticipatory anxiety are essential to mitigate this effect. Drug safety in the context of generic substitution requires robust pharmacovigilance systems. Although studies demonstrate equivalence in efficacy and safety, continuous monitoring of adverse reactions and therapeutic discontinuations associated with formulation changes remains a priority (Bishop et al., 2024). Health systems should implement standardized protocols to document and analyze clinical outcomes when patients transition between generic and brand-name formulations, generating real-world evidence to complement clinical trial data.

Pharmacoeconomic considerations extend beyond simple acquisition cost analysis. The savings from using generics can be redirected to expand complementary mental health services, such as psychotherapy, psychosocial rehabilitation programs, and prevention. This systemic perspective shifts the discussion about generics from a narrow focus on drug substitution to a comprehensive strategy for optimizing healthcare resources (Desai et al., 2019). The COVID-19 pandemic has also highlighted the fragility of pharmaceutical supply chains and the importance of maintaining multiple suppliers of essential medicines. The generic market, by encouraging competition among manufacturers, contributes to the resilience of the pharmaceutical system and reduces vulnerabilities to production or distribution disruptions (Johnston and Holt, 2014). This health security dimension provides an additional argument in favor of policies that actively promote the availability and use of generic medicines for mental health.

Generic medicines represent a transformative tool for democratizing access to mental health treatments, supported by clear scientific evidence of therapeutic bioequivalence and significant economic benefits. However, realizing their potential requires coordinated actions, including evidence-based professional education, public awareness campaigns, enabling regulatory policies, and strong pharmacovigilance systems. Overcoming psychological, economic, and institutional barriers is not just a technical necessity but a matter of health justice that determines whether millions of people with anxiety and depression can access effective treatments without compromising their economic stability. The time to transform the mental health system into a more inclusive and equitable one is now, and generic medicines are an essential part of this transformation.
